# Supplementary material for: Targeting of Protein Kinase CK2 in Acute Myeloid Leukemia Cells Using the Clinical-Grade Synthetic-Peptide CIGB-300
Source: Biomedicines. 2021 Jul 1;9(7):766. doi: 10.3390/biomedicines9070766 (PMC8301452; doi:10.3390/biomedicines9070766)
Supplement: Supplementary file 1 [file biomedicines-09-00766-s001.zip › Supplementary Figures.pdf]

Supplementary Figures

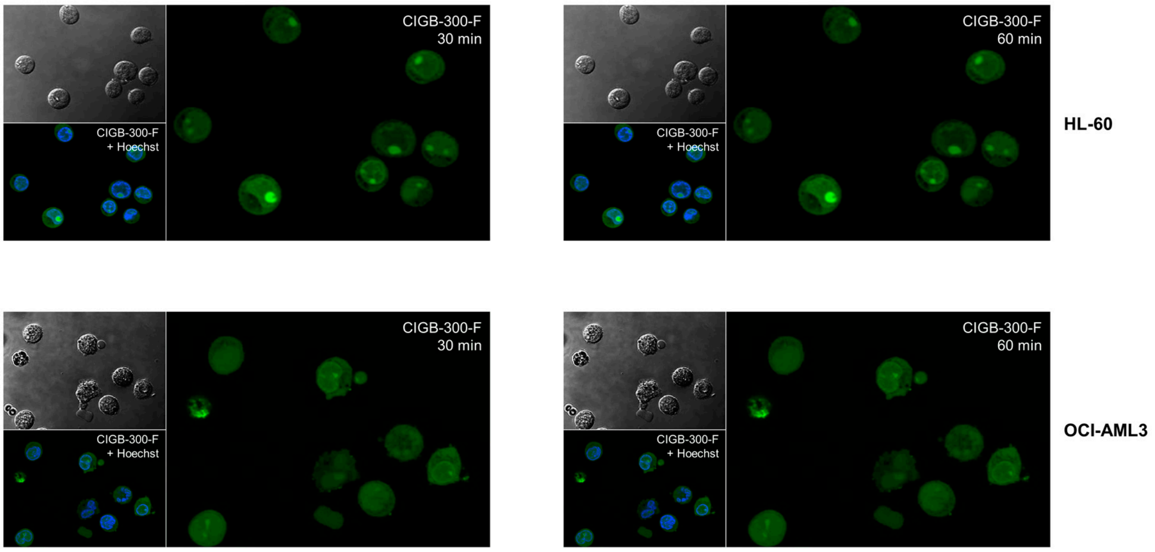

Figure S1. Subcellular distribution of CIGB-300 in AML cells after 30 and 60 min of incubation with 30 μM of CIGB-300-F. A total of 5 optical fields were examined for each experimental condition in confocal microscopy experiment.

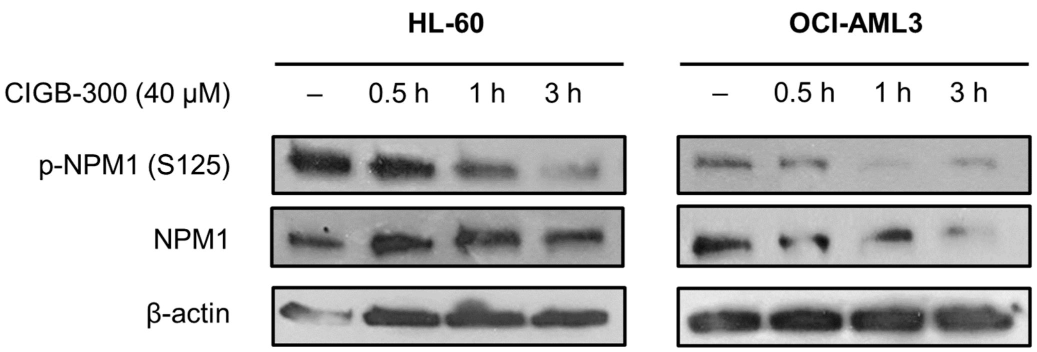

Figure S2. CIGB-300 peptide impairs CK2-mediated phosphorylation of NPM1 in AML cells. Cells treated with 40 µM of CIGB-300 during the indicated times were analyzed by western blot using phospho-specific and total NPM1 antibodies.

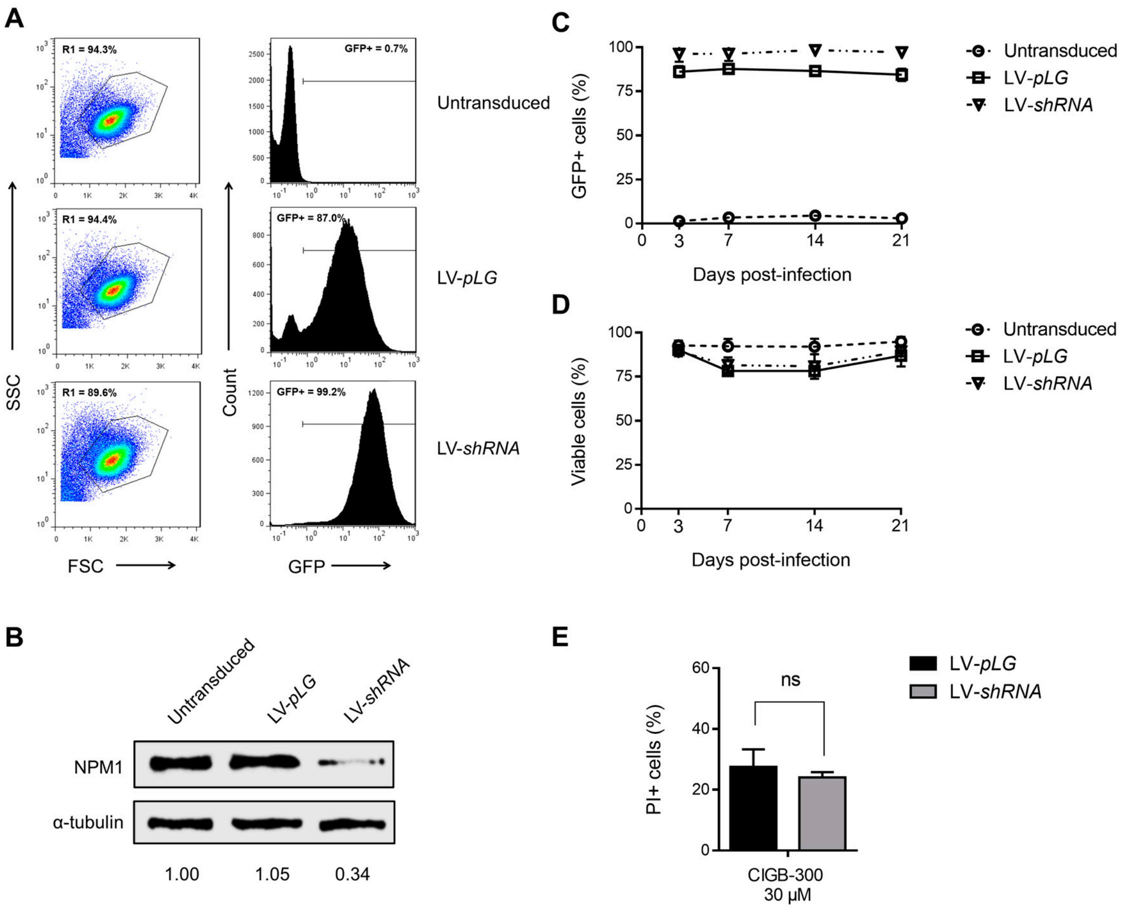

Figure S3. Lentiviral vector-mediated knock-down of NPM1 in THP-1 cells.: (**a**) Flow cytometry analysis of GFP expression in transduced cells at day 7 post-infection. Percentage of GFP+ cells was determined on live-cell population (R1 gate in dot plots); (**b**) Immunoblots from HL-60 transduced cells showing NPM1 protein knock-down in LV-*shRNA* infected cells; (**c**) GFP expression levels and (**d**) viability of transduced THP-1 cells were followed by flow cytometry during three weeks post-infection; (**e**) Sensibility of transduced cells toward the cytotoxic effect of CIGB-300 was evaluated by PI staining. Cells were incubated with 20 or 40 μM of CIGB-300 for 5 h, stained with PI solution and then analyzed by flow cytometry. Results from (**c**), (**d**) and (**e**) are shown as mean ± SD, n = 3. (ns) not significant.
