# Supplementary material for: Targeting of Protein Kinase CK2 in Acute Myeloid Leukemia Cells Using the Clinical-Grade Synthetic-Peptide CIGB-300
Source: Biomedicines. 2021 Jul 1;9(7):766. doi: 10.3390/biomedicines9070766 (PMC8301452; doi:10.3390/biomedicines9070766)
Supplement: Supplementary file 1 [file biomedicines-09-00766-s001.zip › Table S1.pdf]

Table S1: Characteristics of AML patients.

Patient	Sex	Age	AML FAB subtype	Bone marrow blast
1	Female	76 years	FAB-M4	50 %
2	Male	54 years	FAB-M2	80 %
3	Male	59 years	FAB-M2	50 %
4	Female	73 years	FAB-M5	90 %
5	Male	47 years	FAB-M2	60 %
